# Influence of Fiber Orientation on the Water and Ions Transportation of Engineered Cementitious Composite (ECC)

**DOI:** 10.3390/ma16134877

**Published:** 2023-07-07

**Authors:** Abdullah M. Tawfek, Zhi Ge, Jian Li, Kangkang Zhang, Nengdong Jiang, Yingxuan Shao, Yifeng Ling, Branko Šavija

**Affiliations:** 1 School of Qilu Transportation, Shandong University, Jinan 250002, China; d2018009@mail.sdu.edu.cn (A.M.T.); zhige@sdu.edu.cn (Z.G.); jiangnengdong@mail.sdu.edu.cn (N.J.); 202115391@mail.sdu.edu.cn (Y.S.); 2 School of Civil Engineering, Faculty of Engineering, Sana’a University, Sanaa 12544, Yemen; 3 Shandong Hi-Speed Group Co., Ltd., Jinan 250098, China; sdgsrp@163.com; 4 China Construction Industrial & Energy Engineering Group Co., Ltd., Nanjing 210023, China; zhangkangkang4134@cscec.com; 5 Microlab, Faculty of Civil Engineering and Geosciences, Delft University of Technology, 2628 CN Delft, The Netherlands; b.savija@tudelft.nl

**Keywords:** engineered cementitious composite (ECC), fiber orientation, water absorption, sorptivity, sulfate attack, chloride penetration

## Abstract

An engineered cementitious composite (ECC) belongs to a type of high-performance fiber-reinforced materials. Fiber alignment causes the anisotropy of such materials. Herein, the influence of the fiber orientation on water and ion penetration into an ECC was studied. Fiber alignment was achieved using an extrusion approach. Water absorption, sorptivity, chloride penetration resistance, sulfate attack resistance, and freezing–thawing resistance of specimens with fiber aligned horizontally (AH), vertically (AV), and randomly (R), corresponding to the direction of the exposure surface that was studied. The results showed that fibers oriented perpendicular to the water path delayed water migration into the ECC matrix. The sorptivity was significantly affected by the fiber direction. The sorptivity of the AH specimens was 35% and 13% lower than that of the AV and R specimens, respectively. After 180 days of exposure, the chloride penetration depth of the AH specimens was 5.7 mm, which is 13.6% and 20.8% lower than that of the AV and R specimens, respectively. The sulfate ingress profile indicates that the fiber–matrix interface oriented perpendicular to the penetration path can effectively delay sulfate migration. The fiber orientation also influences the compressive strength gain under immersion conditions (Na_2_SO_4_ solution, Na_2_SO_4_ + NaCl solution, and water). Compared with the AH and R specimens, the AV specimens are more sensitive to the immersion condition. In contrast, the fiber orientation has no significant effect on ECC specimens under freeze–thaw cycles. These findings indicate that controlling the fiber alignment and orientation in an ECC can improve its durability under certain exposure conditions.

## 1. Introduction

Three-dimensional concrete printing (3DCP) is a new construction technique that uses automatic control technology to extrude cementitious materials layer by layer [1]. The application of 3D printing technology in the construction industry has received great attention from researchers and industry practitioners. This is due to the fact that 3DCP has the potential to significantly improve concrete construction by reducing construction waste, cost, and time, in addition to increasing safety and geometrical freedom in construction [2,3,4].

An engineered cementitious composite (ECC) belongs to a type of high-performance fiber-reinforced cementitious composite that exhibits high tensile ductility accompanied by the formation of multiple microcracks [5,6,7,8,9]. Generally, an ECC contains cement, fly ash, fine quartz sand, and 2% fiber by volume [10,11,12]. In recent years, many studies have investigated the possibility of developing a 3D-printable ECC (3DP-ECC) [13,14,15]. Promising results, in terms of the mechanical properties of a 3DP-ECC, have been achieved. Zhu et al. [16] found that the flexural strength of 3DP-ECC specimens under a three-point bending test was 4–9% greater than that of mold-cast ECC specimens. The superior flexural strength of 3DP-ECC specimens can be attributed to the aligned fibers along the printing direction. Bao et al. [17] stated that the tensile properties of a printed ECC, including the tensile strength and ductility, are higher than those of a cast ECC by about 4%. Furthermore, the fiber orientation can significantly impact the pore structure and mechanical performance of an ECC [18]. A larger fiber orientation displays a greater snubbing effect, increasing the fiber bridging stress. It is also more prone to fiber rupture compared to smaller fiber orientations [19]. According to Soltan and Li [20], a 3DP-ECC with PVA fiber displays a tensile strength of up to 6 MPa and a tensile strain capacity. Kanarska et al. [21] used 3D printing technology to improve the fiber alignment through different nozzle diameters. The authors’ previous work [22] showed the anisotropy of the mechanical properties of an ECC as a result of the fiber alignment. The smaller the fiber orientation angle is relative to the tensile loading direction, the higher the mechanical properties of the cementitious composite are. In addition, Koker et al. [23] demonstrated that using the extrusion casting method for cementitious composites leads to low matrix porosity. It also promotes the fiber alignment, where the short fibers are aligned in the extrusion direction. Consequently, the mechanical properties, crack development control [24], and water permeability [25] of the cementitious composite are improved.

The addition of fibers in cementitious composites may create more pores and channels, facilitating water transport into the matrix [26,27]. Ramezanianpour et al. [28] stated that the porosity of cementitious composites increases with the fiber content, resulting in a lower resistance to ion penetration in the matrix. Hossein et al. [29] found that the water absorption of cementitious composites increases as the fiber content increases. Akid et al. [30] studied the effect of the fiber content on the chloride permeability of fiber-reinforced concrete. The results indicated that the chloride permeability of an ECC with 0.18% polypropylene fiber is higher than that of one with 0.12% polypropylene fiber. Zhang et al. [31] investigated the effect of steel fiber on the water transport of 3D-printed concrete through sorptivity tests. They concluded that printed specimens transport less water than molded specimens due to the fiber alignment being in the printed direction, which indicated that the fiber orientation had a great effect on the water transport behavior. However, limited studies were dedicated to exploring such effects for an ECC.

In this paper, the effect of the PVA fiber orientation on the water and ion transportation in an ECC was investigated. Extrusion casting was used to enforce the fiber alignment relative to the flow direction. Water absorption, sorptivity, chloride penetration, sulfate attack, and freezing–thawing tests were conducted. This study provided insights into improving ECC durability in practical applications by considering fiber orientation during ECC design.

## 2. Experimental Procedure

### 2.1. Raw Materials and Mixture Proportion

The mixture materials consisted of ordinary Portland cement, fly ash (class F), quartz sand, viscosity modifying admixture (VMA), superplasticizer, water, and polyvinyl-alcohol (PVA) fiber. The chemical compositions of cement and fly ash were tested by X-ray fluorescence (XRF), as shown in Table 1. The quartz sand had a grain size of 80–120 µm. Hydroxypropyl methylcellulose-based VMA was utilized to improve the fiber dispersion. Polycarboxylate type superplasticizer was used to modify the mixture flowability. PVA fibers were supplied by the Kuraray Corporation, Tokyo, Japan. Fly ash and cement were used as the binders. The water–binder ratio (w/b) was 0.26. A common mix proportion from our previous experimental work [22] was used for the ECC mixture, as listed in Table 2.

### 2.2. Mixing and Casting

**Figure 1:**
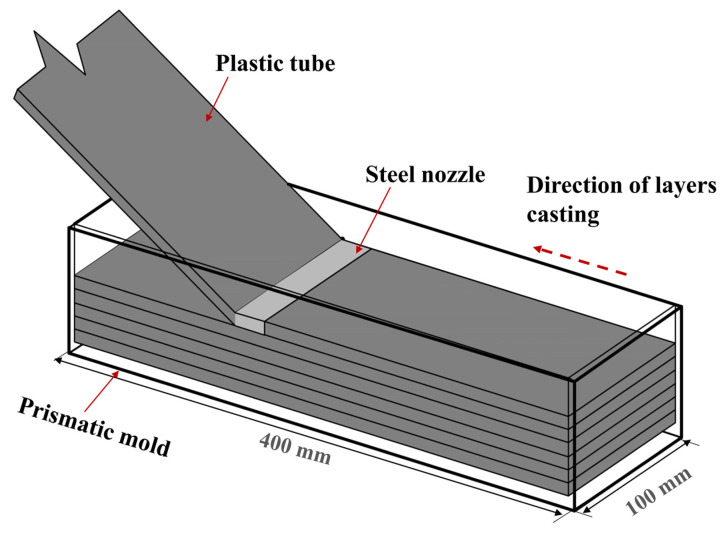
Schematic description of casting a prismatic specimen layer by layer.

ECC mixture was made following the same mixing protocol for all tested specimens. A detailed description of the mixing process is available in our previous work [24]. After mixing, the fresh ECC mixture was cast into a mold (400 × 100 × 100 mm) using two different casting methods (i.e., the extrusion and conventional methods). The extrusion casting method was used in the authors’ previous work [22] to control the fiber orientation. In the conventional method, the mixture was manually poured into the molds, resulting in (presumably) random fiber orientation. In the extrusion method, ECC mixture was poured using an extrusion machine. A plastic tube with a 100 mm diameter was connected to the extrusion machine on one side. A nozzle with a cross section of (100 × 10 mm) was connected to the other side of the tube to force the fibers to preferentially align in the flow direction. The mixture was then extruded in layers with a thickness of 10 mm (see Figure 1).

All specimens were then leveled off, covered with a plastic film to avoid water evaporation, and then demolded after 24 h. Afterward, the specimens were kept in a curing room at temperature of 23 ± 3 °C and relative humidity of 98 ± 2% until the testing date.

**Figure 2:**
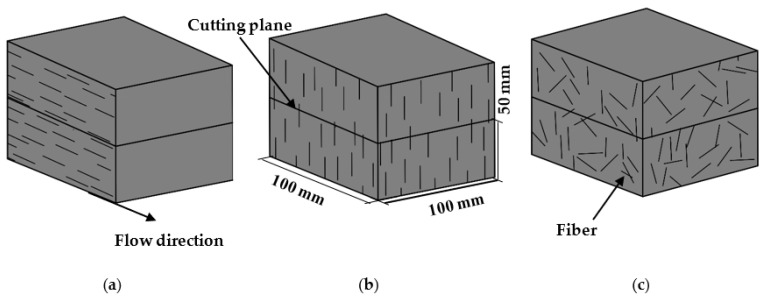
Schematic diagram of various fiber orientations: (**a**) aligned horizontally; (**b**) aligned vertically; (**c**) randomly distributed.

After 28 days of curing, specimens with size of 100 × 100 × 50 mm were cut from each prismatic specimen and used for the durability tests. Three different fiber distributions of ECC specimens were prepared: AH, AV, and R specimens, where AH specimens indicate that the fiber was aligned horizontally with the flow direction, AV specimens indicate that the fiber was aligned vertically with the flow direction, and R specimens indicate that the fiber was randomly distributed (see Figure 2). The specimens were then dried under room conditions for 3 days. To allow one-dimensional ingress, five sides of the specimen were sealed with epoxy resin, leaving only one surface exposed to external conditions. Water absorption, sorptivity, chloride penetration, sulfate attack, water immersion, and combination of chloride and sulfate were all tested. To avoid concentration changes in the external solution due to moisture evaporation during testing, all tested specimens under immersion conditions were conducted in the curing room at 23 ± 3 °C and 98 ± 2% RH. Three specimens were tested for each condition.

## 3. Test Program

**Figure 3:**
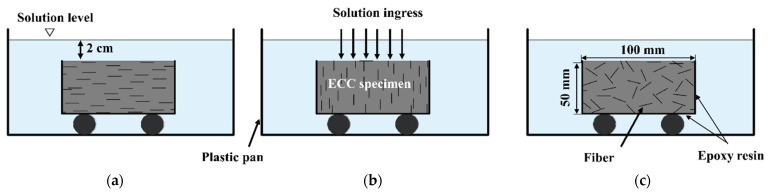
Schematic diagram of ECC specimen under immersion condition: (**a**) fiber aligned horizontally with the exposure surface; (**b**) fiber aligned vertically with the exposure surface; (**c**) fiber distributed randomly.

**Figure 4:**
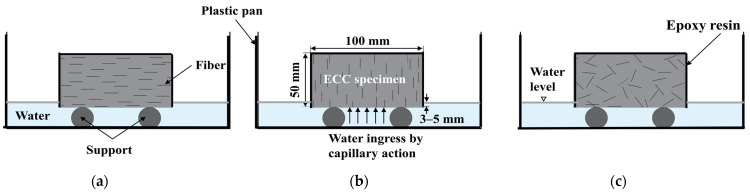
Schematic diagram of ECC specimen under capillary action: (**a**) fiber aligned horizontally with the exposure surface; (**b**) fiber aligned vertically with the exposure surface; (**c**) fiber distributed randomly.

The effect of the fiber orientation on the durability of the ECC was investigated using two different boundary conditions. Water absorption, chloride penetration, and sulfate attack were all performed under immersion conditions (see Figure 3), while a sorptivity test was carried out using capillary action (see Figure 4).

### 3.1. Water Absorption

The absorption test was carried out based on ASTM C642 [32] to determine the absorption ratio and voids ratio in the hardened concrete. The specimens were first oven-dried at 100–110 °C. The specimens were then taken out and cooled at a room temperature of 20–25 °C. Afterward, the mass was measured. The specimens were then completely submerged in distilled water and weighed every 24 h to measure the increase in the mass. Prior to the weight measurement, a dry cloth was used to remove the water from each specimen’s surface. The measurement was completed when the mass difference between two consecutive weight measurements was less than 0.5%, which is known as the saturation phase. The water absorption (*WA*) was calculated using Equation (1).

(1) $$WA(\%)=\frac{m_{1}-m_{2}}{m_{0}}\times 100$$

where *m*_1_ is the specimen weight after immersion in water in (g), and *m*_0_ is the specimen weight before immersion in water in (g).

### 3.2. Water Sorptivity

The rate of water absorption (sorptivity) was measured to investigate the effect of the fiber orientation on the capillary suction. The test was carried out in accordance with ASTM C 1585 [33]. The specimens were cured for 21 days before being immersed in water for 7 days. Afterward, the specimens were cut and sealed, as described in Section 2.2. All specimens were oven-dried at 50 ± 5 °C and then weighed every 24 h. When the difference in mass between the two consecutive measurements was less than 0.1%, the last measurement was defined as the initial mass of the specimen, *m*_0_. The specimens were moved to the tank and placed on the supports. The water level was kept not more than 3–5 mm above the bottom surface of the specimen to avoid water head pressure during the test (see Figure 4).

The test was carried out in two cycles at 23 ± 2 °C. In the first cycle, the specimens were placed in contact with water at intervals of (1, 3, 5, 10, 15, 20, 25, 30, 60, 120, 180, 240, 300, and 360 min). This was followed by subsequent measurements at intervals of 1, 2, 3, 4, 5, 6, and 7 days as the second cycle, which allows the water to be absorbed through capillary suction. The specimens’ weight was recorded after each period, *m*_i_, to determine the initial and final water absorptions, respectively.

The sorptivity coefficient (*S*) can be computed by Equation (2)

(2) $$S=\frac{I}{\sqrt{t}}$$

(3) $$I=\frac{\Delta m}{AD}$$

where *S* is the water sorptivity in mm, √*t* is the square root of the elapsed time in min., *I* is the cumulative absorption, $\Delta m=m_{0}-m_{i}$ is the specimen weight change in (g), *A* is the specimen’s exposed surface area to water in (cm^2^), and *D* is the water density in (g/cm^3^).

### 3.3. Chloride Penetration

**Figure 5:**
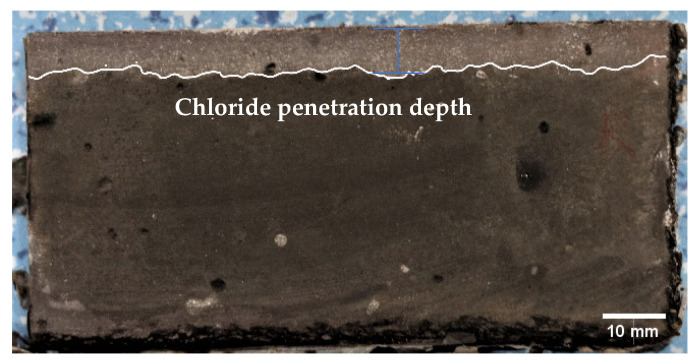
The measurement of chloride penetration depth.

To investigate the effect of the fiber orientation on the resistance to chloride penetration, 3% NaCl solution was used. The sealed specimens were immersed into the solutions for 60, 120, and 180 days under laboratory conditions. The specimens were cut off perpendicular to the exposed surface at each specific time. Afterward, the cut specimen was sprayed with 0.1 mol/L AgNO_3_ solutions to visibly highlight the chloride-contaminated area, as suggested by [34]. After 15 min of stabilization, the white area represented the chloride formed by AgCl, whereas the brown area indicated the absence of chloride due to the formation of Ag_2_O (see Figure 5). The white area was divided into 10 equivalent segments. Each segment’s width should be a minimum of 10 mm [35]. The depth of each segment was measured. The average depth of the white area was selected as the chloride penetration depth. The average chloride penetration depth of three specimens for each method was selected.

### 3.4. Sulfate Attack

**Figure 6:**
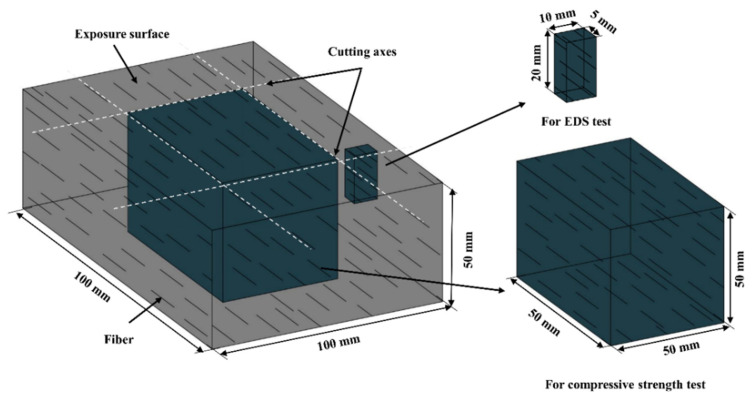
Schematic diagram of specimen used for analysis.

For the sulfate attack, sealed specimens with different fiber orientations were immersed in 5% Na_2_SO_4_ solution and 3% NaCl + 5% Na_2_SO_4_ solution for 180 days, as recommended by Liu et al. [36]. Afterward, a cubic specimen with a length of 50 mm was cut from the specimen and air-dried for 24 h for a compressive strength measurement. In addition, prism specimens with the size of 20 × 10 × 5 mm were selected, scraped flat using a diamond saw, and then well-polished to determine the sulfate depth using energy-dispersive X-ray spectroscopy (EDS) analysis, as shown in Figure 6. The EDS technique is a semi-quantitative analysis used to detect the chemical elements present in a specimen at different concentrations. SEM instrumentation is typically equipped with an EDS system to allow for the chemical analysis of the specimen [37]. The sulfate concentration profile is measured by taking the value of the sulfur intensity at different depths of the specimen, starting at a depth near the exposed surface by EDS, expressed as sulfur wt.%.

**Figure 7:**
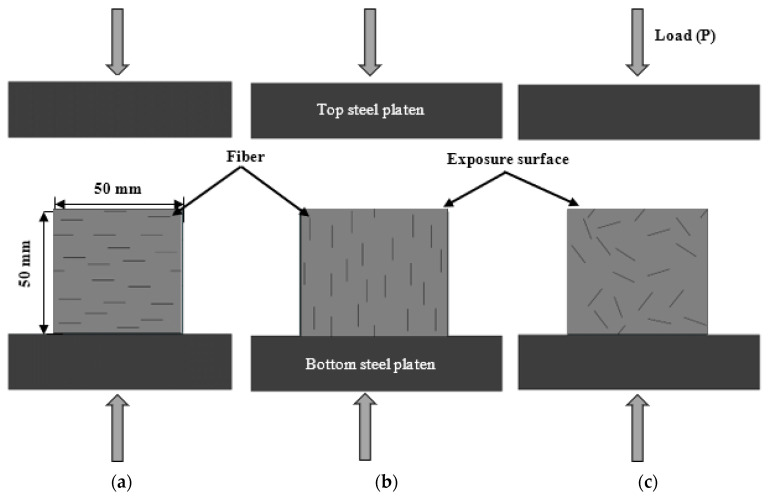
Schematic diagram of compressive test: (**a**) AH, (**b**) AV, and (**c**) R specimens.

For comparison, ECC specimens with different fiber orientations were immersed in water for the same period. The compressive strength of 50 mm cubic specimens after long-term immersion (180 days), as recommended by [38], was measured using a universal testing machine with a loading rate of 0.5 mm/min. The loading direction was the same as the exposure direction. Steel plates were used for the loading (see Figure 7). The average value and standard deviation were calculated from a set of three replicates.

The compressive strength loss rate (*C*,%) was determined according to Equation (4)

(4) $$C=\frac{C_{0}-C_{t}}{C_{0}}\times 100$$

where *C_t_* is the compressive strength (MPa) of the cube specimen measured after 180 days of immersion in Na_2_SO_4_ solution, and *C*_0_ is the compressive strength (MPa) of the control sample measured after 180 days of immersion curing in distilled water. 

### 3.5. Freeze–Thaw Resistance

To evaluate the effect of the fiber orientation on the frost resistance of the ECC, a freezing–thawing (F–T) cycle test based on ASTM C666 [39] was conducted. The surface scaling of the specimens subjected to the freezing and thawing cycles was determined by calculating the change in mass weight (*MC*). The internal damage caused by the freezing and thawing cycles was measured using the resonant frequency method. Since the entire specimen was immersed in water during the F–T test, the conditions of the AH and AV specimens are the same. Thus, only the AH and R specimens were considered for the freeze–thaw test. Three prismatic specimens of 100 × 100 × 400 mm were prepared for each degree of the fiber alignment. All specimens were cured in the curing room for 24 days under standard conditions before being submerged in water for four days. Before testing, the initial dynamic modulus of the elasticity and the mass of the ECC specimens were measured, which were considered as reference. The temperature range of the F–T chamber was set between −18 and 5 °C. Then, all specimens were placed into the F–T chamber. The water surface level inside the F–T chamber was kept 20 mm higher than the top surface of the specimen. At the end of each 50 cycles, each specimen’s surfaces were carefully wiped, and the specimen’s mass was measured. Afterward, the mass loss of the ECC specimens was calculated using Equation (5). The dynamic elastic modulus of each specimen was then measured every 50 cycles up to 300 cycles. The average mass loss and relative dynamic elastic modulus for each method were selected for three specimens.

(5) $$MC(\%)=\frac{w_{1}-w_{0}}{w_{0}}\times 100$$

## 4. Results and Discussion

### 4.1. Water Absorption

**Figure 8:**
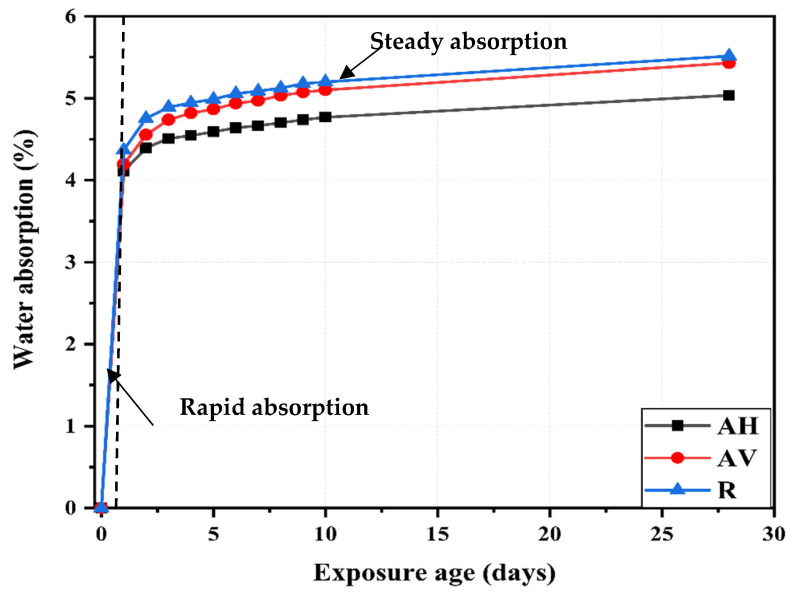
Water absorption of ECC specimens with different fiber orientations.

Figure 8 shows the water absorption capacity of the ECC specimens with different fiber orientations over 28 days. The water absorption capacity curves can be divided into two stages: rapid absorption and steady absorption. In the first stage, the water is rapidly absorbed over time, indicating that the water absorption capacity in this stage is primarily governed by the capillary pores [40]. At the end of this stage, the capillary pores are saturated with water. Afterward, the water continues to transmit into the gel pores, which is defined as the second stage and is mainly controlled by the mechanism of water diffusion [40].

At the end of the first stage (see Figure 8), the water absorption capacity is 4.1%, 4.2%, and 4.4% for the AH, AV, and R specimens, respectively, whereas the water absorption capacity at the end of the second stage is 5.04%, 5.43%, and 5.51% for the AH, AV, and R specimens, respectively. The lower water absorption capacity of the AH and AV specimens can be attributed to the reduction in the internal connectivity of voids and matrix porosity caused by the extrusion casting method.

The water transport occurs via the capillary action along the porous fiber–matrix interface [41,42]. When the fiber is oriented toward the water path, the water penetration process is significantly accelerated throughout the fiber–matrix interface. On the other hand, when the fiber is oriented perpendicular to the water path, water is deposited in this area, which delays the water penetration into the matrix. In the case of a random fiber distribution, the fiber can entangle to create mortar-rich zones, resulting in the formation of voids [43].

After 10 days, the AH and AV specimens absorbed 36.5 g and 37.3 g of water, respectively, while the R specimens absorbed 43.9 g. It is evident that the AH specimens absorb much less water than the AV and R specimens. Even after 28 days immersion, this trend still holds. The reduction in the AH specimens can be attributed to the influence of the fiber direction on delaying the water ingress.

### 4.2. Water Sorptivity

**Figure 9:**
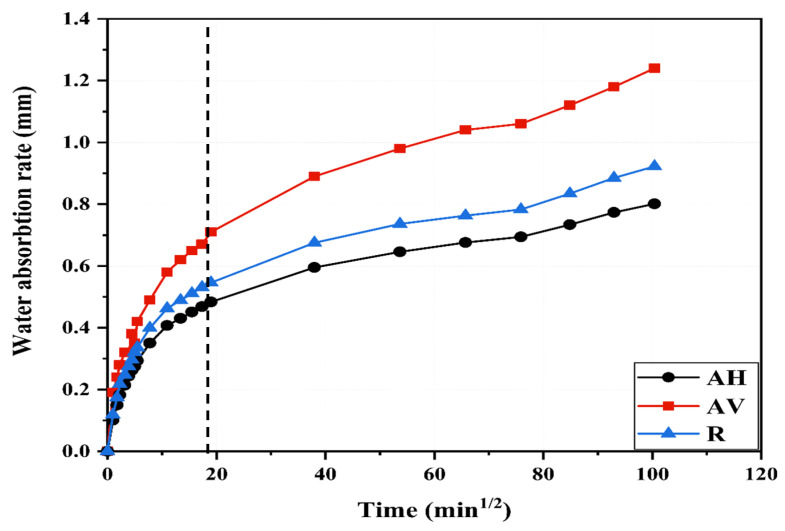
Water absorption rate of ECC specimens at different fiber inclination angles.

Figure 9 presents the water absorption rate as a function of the square root of time. The water absorption rate increases with the square root of time for all specimens. The cumulative water absorption rate rapidly increases due to the capillary action induced under pressure in the unsaturated pores near the specimens’ surface [44]. This rate decreases with the exposure time as the saturation degree of the pore increases.

Since the fiber direction of the AV specimens is parallel to the water penetration path, the initial water absorption rate of the AV specimens is the highest. This means that the capillary pores in the AV specimens would be saturated in a short time compared to those in the AH and R specimens. After 6 h, the water absorption rate of the AV specimens is 0.71 mm, which is 54.6% and 30% higher than that of the AH and R specimens, respectively. This tends to confirm that the fiber being parallel to the water path facilitates water penetration through the fiber–matrix interface, whereas the fiber being perpendicular to the water path (the AH specimens) leads to the delay of water migration, resulting in a decrease in water sorptivity.

Furthermore, the increment of the water absorption rate from 6 h (end of the first cycle) to 7 days (end of the second cycle) is 0.31 mm in the AH specimens, while it is 0.38 mm and 0.53 mm in the R and AV specimens, respectively.

**Figure 10:**
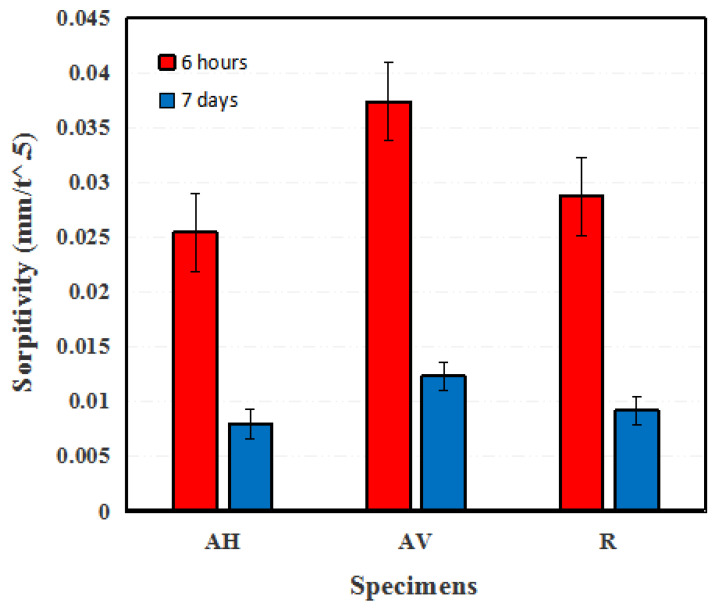
Sorptivity values of ECC specimens with different fiber orientations.

Figure 10 presents the calculated sorptivity of the ECC specimens with different fiber orientations, namely, the AH, R, and AV specimens, at 6 h and 7 days. After 7 days, the sorptivity of the AH specimens is 0.008 mm/t^0.5^, which is 13% and 35% lower than that of the R and AV specimens, respectively. Therefore, lower permeability is expected for the AH specimens [45]. These findings show that the fiber direction influences water migration into the ECC matrix. Note that different trends for sorptivity (Figure 9) and water absorption (Figure 8) are found. Sorptivity is mainly governed by capillary suction [46,47,48], while in immersed conditions, the gravity or pressure gradient also contributes to the water transportation process [49,50].

### 4.3. Chloride Penetration

**Figure 11:**
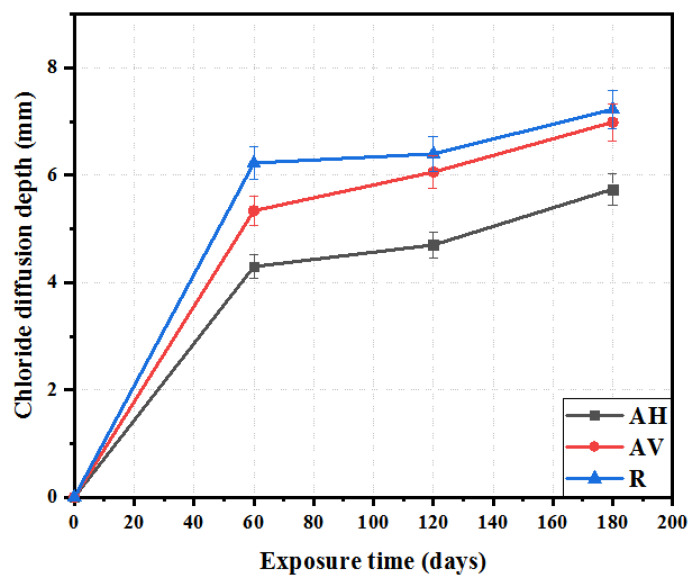
Chloride penetration depth of ECC at different time immersion in 3% NaCl solution.

Figure 11 presents the chloride penetration depth of the ECC specimens with different fiber orientations. The depth of chloride penetration increases with the exposure period (see Figure 11). Much of the increase is in the first 60 days. This accounts for about 80% of the increase after 180 days. This can be attributed to the ongoing hydration of the cement and fly ash, which reduces the porosity, thus delaying the penetration rate of the chloride ions [34,51], in addition to the effect of capillary absorption [52]. After 180 days of exposure, the chloride depth is 5.7 mm, 6.6 mm, and 7.2 mm for the AH, AV, and R specimens, respectively. The reduction in chloride penetration depth of the AH and AV specimens can be ascribed to the densified microstructure due to extrusion. It is worth noting that the AH specimens show a much lower penetration depth than the AV specimens, which tends to confirm that the chloride penetration is governed by the fiber orientation when the matrix has the same density. Šavija et al. [53] observed a similar trend, in which chloride ion penetration occurs parallel to the steel/concrete interface.

### 4.4. Sulfate Attack

**Figure 12:**
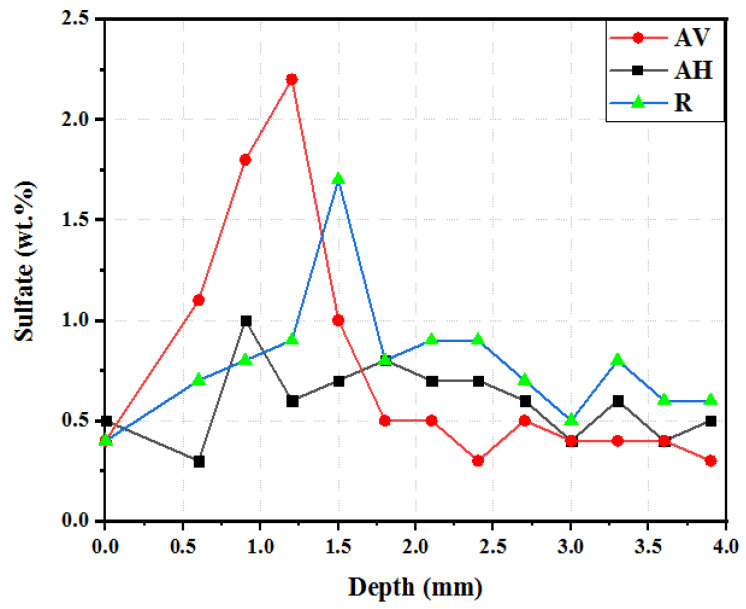
Sulfate profile of ECC specimens at different fiber orientations after 180 days.

Figure 12 presents the sulfate profile perpendicular to the exposed surface after 180 days. The sulfate ingress profile can be identified in three regions. In the region near the surface, the sulfate content in the matrix is reduced due to leaching at the contact surface [54]. The maximum sulfate corresponds to the formation of ettringite and gypsum, which initially reduces the porosity but may cause microcracking and damage to the concrete matrix with ongoing exposure [36]. Lastly, there is a continuous decrease to the bulk content of 0.2–0.6 wt.% SO_3_. In depths greater than 3 mm, the sulfate is almost constant. The peaks of the sulfate profile vary, with that of the AV specimens being the highest. This can be explained by the sulfate ions penetrating the matrix faster through the interface between the fiber and the matrix [55]. The maximum amount of sulfate in the AH specimens is 0.9 wt.% SO_3_, which is 59% and 47% lower than that of the AV and R specimens, respectively. This suggests that the interface being perpendicular to the penetration path can effectively delay the sulfate migration.

**Figure 13:**
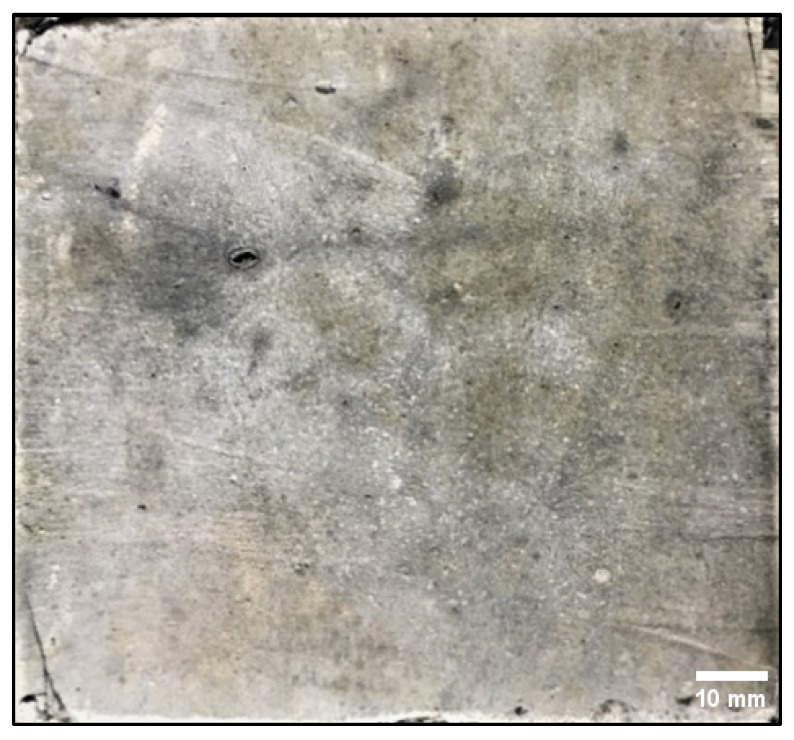
ECC specimen after 180 days of exposure to Na_2_SO_4_.

Figure 13 depicts the surface of the ECC specimen after 180 days of exposure to Na_2_SO_4_ solution. There is no surface cracking observed for any ECC specimens exposed to different conditions. This is because an ECC has a higher tensile resistance, which delays matrix cracking and scaling [36]. The delay in surface cracking kept the diffusivity of the sulfate ions low, preventing accelerated deterioration.

**Figure 14:**
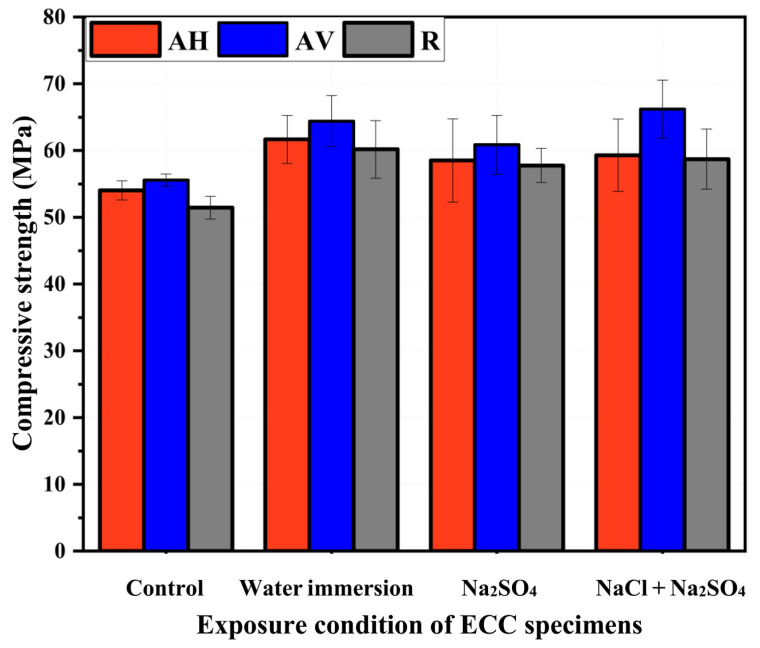
Compressive strength of ECC specimens exposed to Na_2_SO_4_ solution, Na_2_SO_4_ + NaCl solution, and water at 180 days.

The compressive strength of the ECC specimens exposed to Na_2_SO_4_ solution, Na_2_SO_4_ + NaCl solution, and water at 180 days is shown in Figure 14. The compressive strength of the ECC specimens is in the range of 57.76–66.21 MPa. The increment after exposure can be attributed to the continued hydration of the cementitious materials over time. In addition, the ingress of sulfate and chloride ions within the matrix and their interaction with hydration products to form C-S-H gel, ettringite, and Friedel’s salt may contribute to the densification of the microstructure of the ECC, resulting in strength gain [30,36,56].

Regarding the control specimen (28 days curing), the compressive strength of the AV and AH specimens is higher than that of the R specimens in the ECC. This can be attributed to the contribution of the fiber direction to enhance the specimens’ dimensional stability [57]. Under various immersion conditions, the compressive strength of the AV specimens is the highest compared to that of the AH and R specimens (see Figure 14). This can be attributed to the fact that when the fibers are oriented parallel to the load direction, they provide direct resistance to the applied load, enhancing the compressive strength [22]. In addition to the fiber’s ability to confine specimens under uniaxial compressive loading [58], the penetration of sulfate and chloride ions along the porous fiber–matrix interface produces ettringite and Friedel’s salt, which densify the ECC microstructure, increasing the strength [30].

On the other hand, the ECC specimens exposed to Na_2_SO_4_ solution have lower compressive strength than those exposed to water and Na_2_SO_4_ + NaCl solution. The compressive strength of the AV specimens under Na_2_SO_4_ solution is 60.85 MPa, which is 5.5% and 8.1% lower than that of specimens under water and Na_2_SO_4_+ NaCl solution, respectively. Regarding the specimens exposed to Na_2_SO_4_ + NaCl solution, the presence of chloride ions within the sulfate environment mitigates the sulfate attack, reducing the deterioration of concrete [59]. It should be noted that although the compressive strength of the ECC is expected to have a relationship with the absorption capacity and the sorptivity data, the results from these three tests are not in good agreement. The reason is still not clear, which needs further investigation to be figured out.

**Figure 15:**
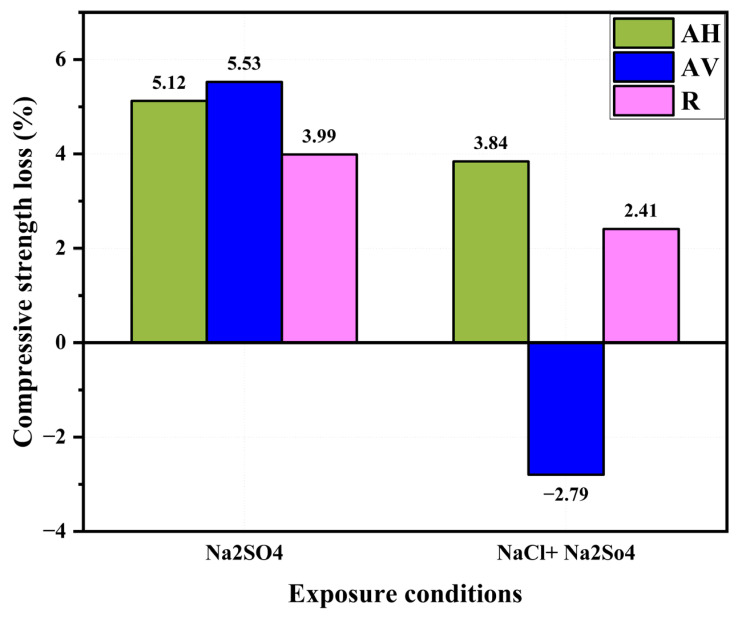
Compressive strength loss rate of ECC specimens after 180 days exposure.

Figure 15 presents the strength loss rate of the ECC specimens after 180 days of exposure. A higher compressive strength loss rate is found for the Na_2_SO_4_ solution’s exposure condition. The negative loss rate of the AV specimens means that they still gain strength under the Na_2_SO_4_ + NaCl solution. This is because of the ongoing formation of Friedel’s salt and ettringite [36,60]. Compared with the Na_2_SO_4_ + NaCl solution-induced degradation, the Na_2_SO_4_ solution-induced degradation is more pronounced. However, to observe a decrease in the mechanical properties, a much higher sulfate solution concentration and a longer exposure period are required.

### 4.5. Freeze–Thaw Resistance

#### 4.5.1. Mass Loss

**Figure 16:**
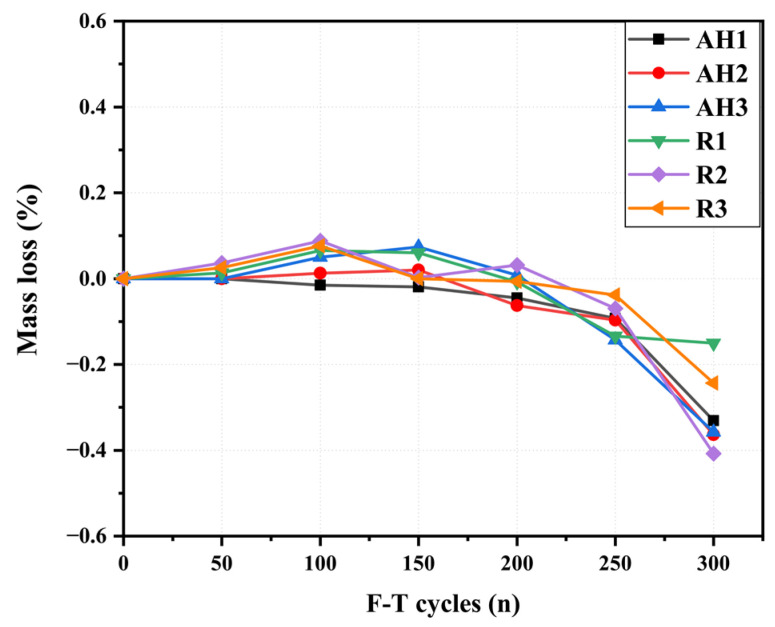
Mass loss of ECC specimens under F–T cycles.

The deterioration of the specimens’ surface under freezing and thawing cycles is evaluated by the computation of the mass loss. Figure 16 shows the mass change and the relative dynamic elastic modulus of the ECC specimens with different fiber orientations. It can be seen that the specimens’ mass increased slightly up to 100 F–T cycles (see Figure 16). The accumulation of freezing water inside specimen voids and the ongoing formation of chemical products could be the cause of the mass gain [61,62,63]. A slight decrease in the specimens’ mass was observed between 100 and 200 F–T cycles. This was followed by a significant decrease in the specimens’ mass between 200 and 300 F–T cycles. The mass loss of the ECC specimens is primarily caused by surface scaling, resulting in fiber exposure [64]. At the end of 300 F–T cycles, very little scaling was observed on the ECC specimens’ surfaces, with an average mass loss of 0.33% for the AH specimens and 0.26% for the R specimens.

**Figure 17:**
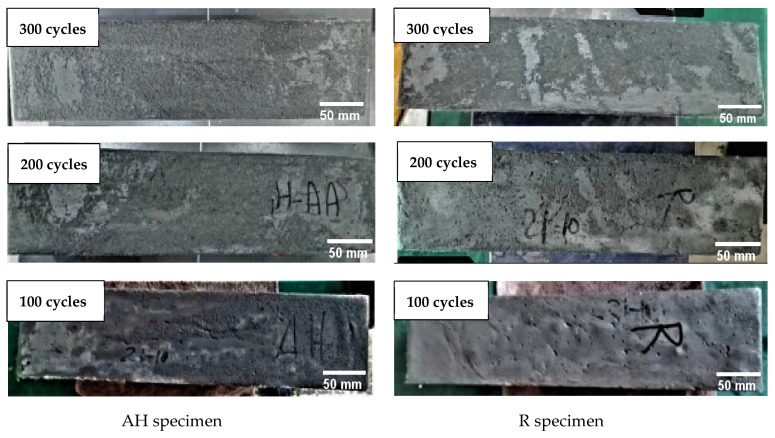
Surface scaling of ECC specimens under F–T cycles.

Based on the visual inspection, the ECC specimens’ surface was smooth for up to 100 cycles. This was followed by the onset of surface scaling after 150 freeze–thaw cycles, which should be carefully removed before continuing on to the next cycle. With the increase in the F–T cycles, the damage on the specimens’ surfaces was mainly the peeling of cement paste. After 300 F–T cycles, more surface scaling of the specimens and more fiber exposure were observed. However, the specimens’ edges were still not degraded (see Figure 17).

#### 4.5.2. Relative Dynamic Elastic Modulus

**Figure 18:**
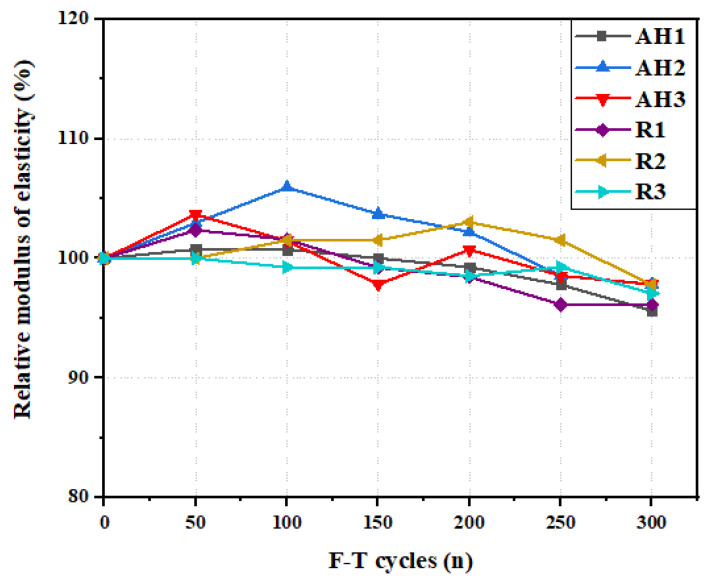
Relative modulus of elasticity of ECC specimens under F–T cycles.

The internal damage of the ECC specimens caused by the freeze–thaw cycles was assessed by the relative dynamic elastic modulus (RDEM). Figure 18 presents the RDEM of the ECC specimens with different fiber orientations. The RDEM values of the ECC specimens showed no noticeable drop during the freeze–thaw cycles. The values ranged from 97% to 105% of the initial value of the RDEM. This can be attributed to the presence of fiber within the ECC matrix, which can significantly increase the connectivity within the ECC matrix [64]. Figure 18 shows that the RDEM of all the ECC specimens increases as the F–T cycles increase up to 50 cycles. This is because of the formation of new hydration products caused by water accumulation during the F–T cycles [61]. Afterward, the RDEM slightly reduces as the number of freeze–thaw cycles increases. This can be attributed to the expansion pressure action caused by the water freezing inside the small matrix pores, resulting in local microcrack formation [65,66]. It should be noted that the RDEM of the ECC was significantly unaffected by the fiber orientation. After 300 F–T cycles, the average relative modulus of elasticity of the AH and R specimens decreased by 2.9% and 3.1%, respectively.

## 5. Conclusions

In the current study, the durability performance of an ECC with different fiber orientations, including water absorption, sorptivity, chloride penetration, and sulfate attack, was evaluated. The following conclusions can be drawn:

- Orienting fibers perpendicular to the water path in the ECC delays water migration into the matrix. After 28 days of immersion, the water absorption capacity of the AH specimens is 5.04%, which is 7.01% and 8.53% lower than that of the AV and R specimens, respectively.
- Fiber direction plays a crucial role in sorptivity. When the fibers are aligned perpendicular to the water path, the sorptivity index of the ECC is clearly reduced, indicating that the oriented-fiber AH specimens can significantly delay sorptivity. After 7 days, the sorptivity of the AH specimens is 0.008 mm/t^0.5^, which is 13% and 35% lower than that of the R and AV specimens, respectively.
- Controlling the fiber orientation improves the chloride resistance of the ECC. The chloride penetration depth of the AH specimens is 5.7 mm, which is 13.6% and 20.8% lower than that of the AV and R specimens, respectively.
- The fibers aligned perpendicular to the penetration path can delay sulfate migration. After 180 days of exposure, the AV specimens show the highest peak in the sulfate profile compared to the AH and R specimens. In addition, all the ECC specimens exposed to the Na_2_SO_4_ solution do not show any surface cracking.
- The AV specimens under the Na_2_SO_4_ + NaCl condition gain more compressive strength than the other specimens, which is 2.8% and 9.5% greater than the specimens under water and the Na_2_SO_4_ solution, respectively.
- The fiber direction has no significant effect on the freezing–thawing resistance of the ECC. A slight difference in the mass loss between the AH and R specimens, 0.07%, is observed.
- The ECC with a controlled fiber alignment could be utilized in applications where moisture resistance is crucial, such as in hydraulic structures, foundations, or coastal environments where chloride exposure is a concern. In future studies, mechanical properties such as ductility, tensile strain capacity, and toughness as a function of fiber orientation under different environmental conditions will be performed for worthwhile applications.

## Figures and Tables

**Table 1:** Chemical compositions of fly ash and cement (%).

Compositions	SiO_2_	Al_2_O_3_	Fe_2_O_3_	CaO	SO_3_	MgO	TiO_2_	MnO	P_2_O_5_	K_2_O
Cement	22.66	6.53	3.05	58.26	4.51	3.00	0.55	0.33	0.13	0.76
Fly Ash	54.59	25.8	6.96	5.49	1.83	0.99	1.26	0.11	0.27	1.76

**Table 2:** Mix proportions of ECC (kg/m^3^).

Cement	Fly Ash	Sand	Fiber Content	Water	Superplasticizer	VMA	w/b
568	682	455	26	325	6.25	0.57	0.26

## Data Availability

The data presented in this study are available on request from the corresponding author.
